# Moderation of the Association Between Individual Food Security and Poor Mental Health by the Local Food Environment Among Adult Residents of Flint, Michigan

**DOI:** 10.1089/heq.2018.0103

**Published:** 2019-06-14

**Authors:** Rachel S. Bergmans, Richard C. Sadler, Julia A. Wolfson, Andrew D. Jones, Daniel Kruger

**Affiliations:** ^1^ Department of Psychiatry, University of Michigan, Ann Arbor, Michigan.; ^2^ Division of Public Health, Michigan State University, Flint, Michigan.; ^3^ Department of Health Management and Policy, University of Michigan, Ann Arbor, Michigan.; ^4^ Department of Nutritional Sciences, University of Michigan, Ann Arbor, Michigan.; ^5^ Population Studies Center, University of Michigan, Ann Arbor, Michigan.

**Keywords:** food insecurity, food environment, social determinants

## Abstract

**Purpose:** Food insecurity is a psychosocial stressor with deleterious effects on mental health. This study examined whether the local food environment moderates the association of individual food insecurity with poor mental health.

**Methods:** Cross-sectional survey data were collected from adult residents of Flint, Michigan (*n*=291), in 2015. Multivariate logistic models assessed whether quality of the local food environment moderated the relationship of food insecurity with poor mental health. A binary indicator of poor mental health was created. Participants were asked to rate their overall “mental or emotional health” using a 5-point Likert scale. Individuals were classified as having either good mental health (i.e., ratings of good, very good, or excellent) or poor mental health (i.e., ratings of fair or poor).

**Results:** In fully adjusted models, food insecurity was associated with 3.2 (95% confidence interval [CI]: 1.6–6.2) times higher odds of poor mental health. However, increased proximate access to vegetables and fruits moderated this association. For example, those in the bottom 25th percentile of access to vegetables had 7.4 (95% CI: 2.7–20.5) times higher odds of poor mental health. In contrast, for those in the top 25th percentile of vegetable access, food insecurity was only marginally associated with poor mental health (odds ratio=2.2; 95% CI: 1.0–4.7).

**Conclusion:** Greater proximate access to vegetables and fruits moderated food insecurity's association with poor mental health. Longitudinal evaluation of programs and policies that improve availability of nutrient-rich foods in food insecure communities is needed to determine whether they yield a mental health benefit.

## Introduction

Poor mental health is among the most debilitating consequences of food insecurity,^1^ including acute psychological pain, anxiety, shame, and depression.^2^ While the link between food insecurity and depression has been demonstrated independent of other socioeconomic resources,^3^ evidence-based strategies that reduce the impact of food insecurity on mental health are not well established. In the United States, roughly 12% of the population faces some level of food insecurity, and rates are even higher—upwards of 20%—among non-white households.^4^ Identifying modifiable community-level characteristics that moderate the influence of food insecurity on mental health could provide an effective policy target and ameliorate health disparities.

Food insecurity is defined as “limited or uncertain availability of nutritionally adequate and safe foods, or limited or uncertain ability to acquire acceptable foods in socially acceptable ways.”^5^ It is a complex experience that encompasses four dimensions at the individual level^6^: inadequate energy intake (i.e., quantity), inadequate nutrient intake (i.e., quality), feelings of deprivation or restricted choice (i.e., psychological acceptability), and abnormal meal patterns such as reducing the size of meals or skipping meals (i.e., social acceptability). Inherent to food insecurity is the contribution of limited resources, financial or otherwise, which is recognized when measuring food insecurity by the United Nations^7^ and the United States Department of Agriculture.^5^ However, food insecurity can exist independent of severe poverty.^4^

A number of underlying pathways may play a role in the influence of food insecurity on mental health. First, food insecurity is associated with diet quality. Food insecure adults consume fewer vegetables, fruits, and dairy products, as well as fewer vitamins and minerals.^8^ Food insecurity has also been associated with the degree to which diet influences immune-inflammatory pathways^9^—considered to play an etiological role in multiple mood disorders, including depression.^10^ Second, specific nutritional deficiencies are also associated with worse mental health.^11^ Finally, in response to food insecurity, persons may be persistently focused on how to obtain necessary food resources. Whether navigating government food assistance programs, relying on food banks, seeking out free food from social networks, or foregoing medical care,^12,13^ appraisal of possible coping strategies can have a psychological burden.^14^

Cohen^15^ has discussed the potential influence of external social, economic, institutional, and community characteristics on individual-level food security status. Limited evidence suggests that low-income areas with poor access to healthy, affordable foods (also known as food deserts) may exacerbate the pathways by which food insecurity influences mental health.^16,17^ Whether the quality of local food environment moderates the relationship of food insecurity with mental health is not well established.

This study examines the extent to which the quality of local food environment moderates the link between food insecurity and mental health among adult residents of Flint, Michigan. Flint offers a unique population for assessing the moderating influence of local food environments on the association between food insecurity and mental health status. Decades of disinvestment in Flint have yielded high rates of poverty and exacerbated other risk factors for poor mental health.^18^ Subsequently, most chain grocery stores have departed from the city. The stores that remain have foods of poorer quality and less variety.^19^ Furthermore, since 2014, there have been observed increases in stress and anxiety due to residents learning that city water was contaminated with unsafe lead levels^20^—infamously known as the Flint Water Crisis.^21^

It is hypothesized that the association of food insecurity with poor mental health will be weaker among those who have greater proximate access to nutrient-dense food groups. In particular, given that fruits and vegetables are rich in anti-inflammatory micronutrients,^22^ local availability of fruits and vegetables is expected to attenuate the relationship of food insecurity with poor mental health.

## Methods

### Sample

The Speak to Your Health (STYH) survey is a biennial cross-sectional survey that provides sociodemographic and health data on adult residents in Flint and Genesee County.^23^ Germane to this article, the 2015 STYH survey provides information on mental health status, food insecurity, subject residential address, and a number of important demographic and socioeconomic factors. Residential address is necessary to derive neighborhood-level food store scores as created in Shaver et al.^19^ Data collection for the 2015 STYH survey was carried out using mailed hard copy, in-person hard copy, and online surveys. Postcard invitations were mailed to a random sample of households representing all residential Census Tracts in the City of Flint. Postcards included the survey website URL containing a link to the online version of the survey. These invitations were followed by a mailed hard copy survey; 40% of respondents were recruited through these means. Participants were also recruited through e-mail lists of local employers and educational institutions (29%), in-person recruitment by community-based organizations and health clinics (13%), and advertisements on local media (9%). Other participants reported being notified by friends or family members (6%) or seeing flyers advertising the survey (3%).

Inclusion criteria for STYH included being a resident of Genesee County and being 18 years or older. Sample demographics of STYH participants were periodically compared to population characteristics and community-based efforts oversampled underrepresented neighborhoods. When compared to U.S. Census population estimates for Flint, Michigan,^24^ the study population had a greater proportion of those who are non-Hispanic white, older than 65 years, and female. However, rates of employment and poverty were similar. Among the 393 STYH participants who resided in Flint, the analytic sample was limited to those with complete case data for variables of interest (*n*=291). No other exclusion criteria were applied.

STYH was approved by the University of Michigan Institutional Review Board for Health and Behavioral Sciences and permission was granted by the STYH survey Committee to use these data for the analyses presented in this study. STYH participants provided informed consent before survey administration.

### Measures

#### Food insecurity

To identify participants who were likely food insecure, participants were asked, “How stable, secure, or reliable is a healthy diet of food in your life?” Subjects who reported a healthy diet of food to be moderately or completely stable, secure, or reliable were classified as not having food insecurity. Any other response (e.g., somewhat secure, equally secure and insecure, and moderately or completely insecure) was coded as food insecurity because even moderate levels of food insecurity can be harmful for health.^25^

#### Poor mental health

A binary indicator of poor mental health was created. Participants were asked to rate their overall “mental or emotional health” using a 5-point Likert scale that included the following response options: (1) excellent; (2) very good; (3) good; (4) fair; and (5) poor. Individuals were classified as having either good mental health (i.e., ratings of good, very good, or excellent) or poor mental health (i.e., ratings of fair or poor).

Poor mental health represents an important health indicator, independent from diagnosed psychopathology. In a scoping review of single-item measures of self-rated mental health, Ahmad et al. observed that such measures are representative of multi-item measures of mental health and associated with health care utilization.^26^ Subjective assessment of general mental and emotional health—as was asked of STYH participants—may lead individuals to consider their sense of purpose and overall life satisfaction, in addition to the presence or absence of negative mental or emotional states.^27^ Furthermore, evidence indicates that single-item measures of poor mental health are associated with mental morbidity, including stress level, depressive symptoms, and major depressive episodes.^28^

#### Local food environment

The quality of local food environment was measured using the Flint Food Store Survey (including the same food categories and points used in the original study).^19^ This survey was created by combining a Nutrition Environment Measures Survey in Stores (NEMS-S) assessment of every food store in and around the City of Flint with spatial analysis, yielding individual store scores and neighborhood-level metrics for healthy food access. The NEMS-S is a valid and reliable food store audit tool that captures quality, variety, and price of foods in several categories, including fruits, vegetables, grains, dairy and alternatives, and meat and alternatives.^29^ Store scores were used in separate kernel density analyses to create surfaces of accessibility for every location in the City of Flint. Kernel density estimation is a nonparametric method commonly used to estimate unknown densities for a given geographic area.^30^ This study used a search radius of 1 mile since this is a common metric for measuring access to grocery stores and other food sources.^31,32^

#### Covariates

Demographic covariates included age group (18 to <40 years, 40 to <65 years, 65 to 93), race/ethnicity (Non-Hispanic white; Non-Hispanic black; other, e.g., Hispanic, Multi-racial, Asian, Native American/American Indian, Hawaiian Native, Pacific Islander, or unknown), sex (male or female), and marital status (married, never married, separated, divorced, or widowed). Socioeconomic covariates included educational attainment (high school degree or less; some college; and college degree or above) and employment status (employed; unemployed; and other, e.g., on disability, student, or homemaker). To measure financial insecurity, participants were asked, “How difficult is it for you to live on your total household income?” Participants were classified into three groups: (1) those who responded difficult, very difficult, or extremely difficult; (2) those who responded somewhat difficult; and (3) those who responded not at all difficult. Finally, the presence of chronic physical illness was also included as a covariate given that chronic conditions have a high comorbidity with depressive disorders,^33^ are often financially burdensome, and are hypothesized to have a bidirectional association with food insecurity.^34^ Presence of a chronic physical illness was coded as “yes” among individuals with a diagnosis history of one or more of the following conditions: high blood pressure, heart disease, stroke, asthma, sleep disorder, sarcoidosis, or sickle cell anemia. Those with no history of these conditions were not considered to have a chronic physical illness.

### Statistical analysis

All analyses were conducted using SAS^®^ version 9.4.^35^ First, *χ*^2^ and type III *F* tests were used to determine the distribution of food insecurity, the local food environment, mental health status, and covariates for the total sample population, and by mental health status and food security status. Next, logistic regression was used to assess a series of models for the association between food insecurity and poor mental health. Model 1 accounted for demographic covariates (age, race/ethnicity, sex, marital and status); Model 2 accounted for all covariates in Model 1 plus socioeconomic indicators (educational attainment, employment status, and financial ability to meet household needs); and Model 3 accounted for all covariates in Model 2 plus the presence of chronic physical illness.

Finally, moderation of the association between food insecurity and poor mental health by five dimensions of the local food environment (vegetables, fruit, grains, dairy, and meat)—derived from individual category scores of the Flint Food Store Survey^19^—was tested using logistic regression by adding interaction terms with food insecurity to Model 3. Initially, only the logit coefficients were reported because when a variable is involved in an interaction, it is not possible to calculate a single odds ratio (OR) estimate.^36^ Thus, to improve interpretation of interaction terms, the odds of poor mental health were calculated by NEMS-S population quartiles for those that reached statistical significance (*α*=0.05).

## Results

Table 1 presents descriptive statistics for the analysis sample. Among study participants, 22% had poor mental health. Poor mental health was more common among middle aged adults (75%) than elderly adults (10%). The prevalence of poor mental health did not differ by sex or race/ethnicity. Overall, the sample was 30% male, 55% Non-Hispanic white, 30% Non-Hispanic black, and 15% of a different racial/ethnic category. Those with poor mental health were less likely to be married (19% vs. 42%) and more likely to be separated, divorced, or widowed (37% vs. 25%) than those with good mental health. Among those with poor mental health, 62% were food insecure. In contrast, only 29% of individuals with good mental health were food insecure. No differences existed, however, in quality of local food environment by mental health status.

**Table 1.** Descriptive Statistics for Total Sample and by Perceived Mental Health Status, Speak to Your Health 2015 Survey

Characteristics		Mental health		*p*^a^
	Total sample, *n*=291	Poor, *n*=63	Good, *n*=228	
Age group, years, *n* (%)				0.001
18 to <40	74 (25.4)	10 (15.9)	64 (28.1)	
40 to <65	158 (54.3)	47 (74.6)	111 (48.7)	
65 to 93	59 (20.3)	6 (9.5)	53 (23.3)	
Male, *n* (%)	90 (30.9)	20 (31.8)	70 (30.7)	0.874
Race/Ethnicity, *n* (%)				0.533
Non-Hispanic white	165 (56.7)	33 (52.4)	132 (57.9)	
Non-Hispanic black	91 (31.3)	20 (31.8)	71 (31.1)	
Other	35 (12.0)	10 (15.9)	25 (11.0)	
Marital status, *n* (%)				0.004
Married	107 (36.8)	12 (19.1)	95 (41.7)	
Single	105 (36.1)	28 (44.4)	77 (33.8)	
Separated, divorced, or widowed	79 (27.2)	23 (36.5)	56 (24.6)	
Educational Attainment, *n* (%)				<0.001
≥College degree	104 (35.7)	11 (17.5)	93 (40.8)	
Some college	121 (41.6)	28 (44.4)	93 (40.8)	
≤High school degree	66 (22.7)	24 (38.1)	42 (18.4)	
Employment status, *n* (%)				0.005
Employed	126 (43.3)	16 (25.4)	110 (48.3)	
Other	147 (50.5)	42 (66.7)	105 (46.1)	
Unemployed	18 (6.2)	5 (7.9)	13 (5.7)	
Financial ability to meet household needs, *n* (%)				<0.001
Not difficult	79 (27.2)	5 (7.9)	74 (32.5)	
Somewhat difficult	88 (30.2)	17 (27.0)	71 (31.1)	
Extremely difficult	124 (42.6)	41 (65.1)	83 (36.4)	
Comorbidity,^b^ n (%)	201 (69.1)	54 (85.7)	147 (64.5)	0.001
Food insecure, *n* (%)	104 (35.7)	39 (61.9)	65 (28.5)	<0.001
Quality of the local food environment^c^ 1 mile kernel density, mean (95% CI)				
Vegetables	13.6 (12.2–15.0)	15.3 (12.4–18.2)	13.2 (11.5–14.8)	0.220
Fruits	20.9 (18.8–23.1)	23.8 (19.3–28.4)	20.1 (17.7–22.6)	0.166
Grains	49.9 (44.9–54.9)	57.3 (46.0–68.6)	47.8 (42.2–53.4)	0.124
Dairy	21.0 (19.2–22.9)	24.3 (20.0–28.6)	20.1 (18.1–22.1)	0.065
Meat	31.3 (28.4–34.2)	35.8 (29.2–42.4)	30.1 (26.9–33.3)	0.110

^a^ *χ*^2^ or *F* test.

^b^ Diagnosis history of high blood pressure, heart disease, stroke, cancer, diabetes, asthma, sleep disorder, sarcoidosis, or sickle cell anemia.

^c^ NEMS Score, Shaver et al.^19^

CI, confidence interval; NEMS, Nutrition Environment Measures Survey.

Table 2 presents descriptive statistics by food security status. Those who were food insecure were more likely to be young adults, aged 18 to <40 years (32% vs. 22%), non-Hispanic black (40% vs. 26%), and single (46% vs. 31%). Socioeconomic status was also associated with food insecurity—those who were food insecure were more likely to have *less than or equivalent to a* high school degree (30% vs. 19%) and have extreme difficulty meeting household needs (61% vs. 33%). Chronic physical illness status and quality of the local food environment did not differ by food security status.

**Table 2.** Descriptive Statistics by Food Insecurity Status, Speak to Your Health 2015 Survey

Characteristics	Food insecure		*p*^a^
	Yes, *n*=104	No, *n*=187	
Age group, years, *n* (%)			0.021
18 to <40	33 (31.7)	41 (21.9)	
40 to <65	56 (33.0)	53 (41.0)	
65 to 93	58 (55.8)	100 (53.5)	
Male, *n* (%)	36 (34.6)	29 (28.9)	0.310
Race/Ethnicity, *n* (%)			0.006
Non-Hispanic white	46 (44.2)	119 (63.6)	
Non-Hispanic black	42 (40.4)	49 (26.2)	
Other	16 (15.4)	19 (10.2)	
Marital status, *n* (%)			0.017
Married	29 (27.9)	78 (41.7)	
Single	48 (46.2)	57 (30.5)	
Separated, divorced, or widowed	27 (26.0)	52 (27.8)	
Educational attainment, *n* (%)			0.002
≥College degree	24 (23.1)	80 (42.8)	
Some college	49 (47.1)	72 (38.5)	
≤ High school degree	31 (29.8)	35 (18.7)	
Employment status, *n* (%)			0.330
Employed	39 (37.5)	87 (46.5)	
Other	58 (55.8)	89 (47.6)	
Unemployed	7 (6.7)	11 (5.9)	
Financial ability to meet household needs, *n* (%)			<0.001
Not difficult	11 (10.6)	68 (36.4)	
Somewhat difficult	30 (28.9)	58 (31.0)	
Extremely difficult	63 (60.6)	61 (32.6)	
Comorbidity,^b^*n* (%)	72 (69.2)	129 (69.0)	0.965
Mental health status			<0.001
Poor	39 (37.5)	24 (12.8)	
Good	65 (62.5)	163 (87.2)	
Quality of the local food environment^c^ 1 mile kernel density, mean (95% CI)			
Vegetables	15.2 (12.9–17.4)	12.8 (10.9–14.6)	0.107
Fruits	22.4 (19.0–25.9)	20.1 (17.3–22.9)	0.309
Grains	50.3 (42.3–58.3)	49.6 (43.2–56.0)	0.896
Dairy	22.0 (19.1–24.9)	20.5 (18.1–22.9)	0.428
Meat	32.2 (27.7–36.7)	30.8 (27.0–34.5)	0.638

^a^ *χ*^2^ or *F* test.

^b^ Diagnosis history of high blood pressure, heart disease, stroke, cancer, diabetes, asthma, sleep disorder, sarcoidosis, or sickle cell anemia.

^c^ NEMS Score, Shaver et al.^19^

Table 3 presents ORs and 95% confidence intervals (CIs) for the association between food insecurity and poor mental health. Those who indicated food insecurity had higher odds of poor mental health (OR=4.1; 95% CI: 2.3–7.3). When accounting for demographic covariates in Model 1, this association remained unchanged (OR=4.2; 95% CI: 2.2–7.9). When accounting for socioeconomic covariates, however, the magnitude of the association was lower in Model 2 (OR=3.2; 95% CI: 1.6–6.3). Adjustment for the presence of a chronic physical illness in Model 3 did not further attenuate this association (OR=3.2; 95% CI: 1.6–6.2). Other covariates associated with poor mental health in the fully adjusted model (*α*=0.05) included age group, marital status, and presence of a chronic physical illness.

**Table 3.** Odds Ratios and 95% Confidence Intervals for the Association Between Food Insecurity and Poor Mental Health, Speak to Your Health 2015 Survey

	Crude		Model 1^a^		Model 2^b^		Model 3^c^	
	OR (95% CI)	*p*^d^	OR (95% CI)	*p*^d^	OR (95% CI)	*p*^d^	OR (95% CI)	*p*^d^
Food insecurity	4.1 (2.3–7.3)	<0.000	4.2 (2.2–7.9)	<0.000	3.2 (1.6–6.3)	0.001	3.2 (1.6–6.2)	0.001
Age group, years				0.001		0.006		0.026
18 to <40			Ref.		Ref.		Ref.	
40 to <65			3.6 (1.5–8.4)		2.9 (1.2–7.1)		2.0 (0.7–5.2)	
65 to 93			1.0 (0.3–3.5)		0.8 (0.2–2.9)		0.5 (0.1–2.1)	
Male			1.0 (0.5–2.0)	0.966	0.9 (0.4–1.9)	0.766	0.9 (0.4–1.8)	0.697
Race/Ethnicity				0.420		0.506		0.417
Non-Hispanic white			Ref.		Ref.		Ref.	
Non-Hispanic black			0.8 (0.4–1.6)		0.7 (0.3–1.5)		0.7 (0.3–1.5)	
Other			1.5 (0.6–3.9)		1.3 (0.5–3.4)		1.4 (0.5–4.0)	
Marital status				0.007		0.022		0.024
Married			Ref.		Ref.		Ref.	
Single			3.0 (1.3–6.9)		3.0 (1.2–7.3)		3.0 (1.2–7.3)	
Separated, divorced, or widowed			3.5 (1.5–8.2)		3.1 (1.3–7.7)		3.2 (1.3–7.9)	
Educational attainment						0.082		0.086
≥College degree					Ref.		Ref.	
Some college					1.5 (0.6–3.6)		1.6 (0.7–3.7)	
≤High school degree					2.8 (1.1–7.0)		2.8 (1.1–7.2)	
Employment status						0.201		0.334
Employed					Ref.		Ref.	
Other					1.4 (0.4–5.3)		1.3 (0.3–5.1)	
Unemployed					2.0 (0.9–4.4)		1.8 (0.8–4.0)	
Financial ability to meet household needs						0.112		0.158
Not difficult					Ref.		Ref.	
Somewhat difficult					2.0 (0.6–6.5)		2.0 (0.6–6.6)	
Extremely difficult					3.0 (1.0–9.0)		2.8 (0.9–8.5)	
Comorbidity^e^							2.5 (1.0–6.3)	0.048

^a^ Model 1 accounts for demographic characteristics.

^b^ Model 2 accounts for covariates in Model 1+socioeconomic characteristics.

^c^ Model 3 accounts for covariates in Model 2+comorbidities.

^d^ Type III *χ*^2^-test.

^e^ Diagnosis history of high blood pressure, heart disease, stroke, cancer, diabetes, asthma, sleep disorder, sarcoidosis, or sickle cell anemia.

OR, odds ratio.

The association between food insecurity and poor mental health was moderated by access to vegetables (interaction term *p*=0.021) and fruits (interaction term *p*=0.015) (Table 4). The negative interaction terms for both vegetables and fruits indicate a lower magnitude of association between food insecurity and poor mental health, with higher kernel density of vegetable and fruit access. Although tests of moderation by grains, dairy, and meat access showed a similar trend, they did not meet criteria for statistical significance (*α*=0.05). Table 5 demonstrates that the association of poor mental health with food insecurity is lower with greater access to vegetables and fruits. For example, for those in the bottom 25th percentile of vegetable access, food insecurity was associated with 7.4 (95% CI: 2.7–20.5) times higher odds of poor mental health. In contrast, for those in the top 25th percentile of vegetable access, food insecurity was only marginally associated with poor mental health (OR=2.2; 95% CI: 1.0–4.7).

**Table 4.** Parameter Estimates (βs) and Standard Errors for Moderation of the Association Between Food Insecurity and Poor Mental Health by Quality of the Local Food Environment, Speak to Your Health 2015 Survey

	Local food environment^a^ 1 mile kernel density			
	Vegetables		Fruit	
	β (SE)	*p*^b^	β (SE)	*p*^b^
Food insecurity	1.09 (0.29)	<0.000	1.12 (0.29)	<0.000
Local food environment main effect	0.00 (0.02)	0.882	0.00 (0.01)	0.780
Local food environment^*^food insecurity	−0.03 (0.01)	0.021	−0.02 (0.01)	0.015
Age group, years		0.013		0.014
18 to <40	Ref.		Ref.	
40 to <65	0.75 (0.26)		0.75 (0.26)	
65 to 93	−0.70 (0.39)		−0.67 (0.39)	
Male	−0.18 (0.39)	0.635	−0.21 (0.38)	0.591
Race/Ethnicity		0.414		0.455
Non-Hispanic white	Ref.		Ref.	
Non-Hispanic black	−0.36 (0.27)		−0.34 (0.27)	
Other	0.35 (0.34)		0.33 (0.34)	
Marital status		0.025		0.025
Married	Ref.		Ref.	
Single	0.30 (0.26)		0.25 (0.26)	
Separated, divorced, or widowed	0.44 (0.27)		0.49 (0.27)	
Educational attainment		0.042		0.045
≥College degree	Ref.		Ref.	
Some college	−0.07 (0.23)		−0.08 (0.23)	
≤High school degree	0.63 (0.26)		0.63 (0.26)	
Employment status		0.327		0.342
Employed	Ref.		Ref.	
Other	−0.15 (0.45)		−0.18 (0.45)	
Unemployed	0.37 (0.29)		0.37 (0.29)	
Financial ability to meet household needs		0.178		0.173
Not difficult	Ref.		Ref.	
Somewhat difficult	0.07 (0.28)		0.07 (0.28)	
Extremely difficult	0.47 (0.26)		0.48 (0.26)	
Comorbidity^c^	0.86 (0.47)	0.066	0.84 (0.47)	0.072

	Local food environment^a^ 1 mile kernel density					
	Grains		Dairy		Meat	
	β (SE)	*p*^b^	β (SE)	*p*^b^	β (SE)	*p*^b^
Food insecurity	0.95 (0.29)	0.001	1.01 (0.32)	0.001	1.04 (0.31)	0.001
Local food environment main effect	0.00 (0.00)	0.560	0.01 (0.01)	0.556	0.01 (0.01)	0.478
Local food environment^*^food insecurity	−0.01 (0.00)	0.108	−0.02 (0.01)	0.090	−0.01 (0.01)	0.069
Age group, years		0.016		0.014		0.014
18 to <40	Ref.		Ref.			
40 to <65	0.73 (0.26)		0.76 (0.26)		0.75 (0.26)	
65 to 93	−0.65 (0.39)		−0.65 (0.39)		−0.65 (0.39)	
Male	−0.21 (0.38)	0.580	−0.17 (0.38)	0.659	−0.22 (0.38)	0.559
Race/Ethnicity		0.492		0.458		0.480
Non-Hispanic white	Ref.		Ref.			
Non-Hispanic black	−0.32 (0.27)		−0.33 (0.27)		−0.33 (0.27)	
Other	0.33 (0.34)		0.36 (0.34)		0.32 (0.34)	
Marital status		0.029		0.024		0.030
Married	Ref.		Ref.			
Single	0.28 (0.25)		0.30^*^ (0.25)		0.26 (0.25)	
Separated, divorced, or widowed	0.45 (0.26)		0.45^*^ (0.26)		0.46 (0.27)	
Educational attainment		0.068		0.074		0.075
≥College degree	Ref.		Ref.			
Some college	−0.05 (0.23)		−0.06 (0.23)		−0.05 (0.23)	
≤High school degree	0.58 (0.26)		0.57 (0.26)		0.57 (0.26)	
Employment status		0.376		0.385		0.364
Employed	Ref.		Ref.			
Other	−0.09 (0.44)		−0.06 (0.44)		−0.13 (0.44)	
Unemployed	0.32 (0.28)		0.30 (0.28)		0.34 (0.28)	
Financial ability to meet household needs		0.226		0.206		0.243
Not difficult	Ref.		Ref.			
Somewhat difficult	0.10 (0.28)		0.09 (0.28)		0.11 (0.28)	
Extremely difficult	0.42 (0.25)		0.44 (0.25)		0.41 (0.25)	
Comorbidity^c^	0.88 (0.46)	0.058	0.90 (0.46)	0.050	0.87 (0.46)	0.060

Log-odds multivariate linear regression.

^a^ NEMS Score, Shaver et al.^19^

^b^ Joint *χ*^2^-test.

^c^ Diagnosis history of high blood pressure, heart disease, stroke, cancer, diabetes, asthma, sleep disorder, sarcoidosis, or sickle cell anemia.

**Table 5.** Odds Ratios and 95% Confidence Intervals for the Association Between Food Insecurity and Poor Mental Health, Moderated by Dimensions of the Local Food Environment, Speak to Your Health 2015 Survey

Local food environment dimension	Local food environment^a^ 1 mile kernel density					*p*^b^
	Min,^c^ OR (95% CI)	25th percentile,^c^ OR (95% CI)	50th percentile,^c^ OR (95% CI)	75th percentile,^c^ OR (95% CI)	Max,^c^ OR (95% CI)	
Vegetables	8.9 (2.9–27.5)	7.4 (2.7–20.5)	4.2 (2.0–8.7)	2.2 (1.0–4.7)	0.4 (0.1–2.7)	0.021
Fruits	9.5 (3.1–29.2)	6.9 (2.7–17.7)	4.9 (2.2–10.7)	2.4 (1.1–4.9)	0.3 (0.0–2.3)	0.015

ORs account for age group, gender, race/ethnicity, marital status, educational attainment, employment status, financial ability to meet household needs, and comorbidities.

^a^ NEMS Score, Shaver et al.^19^

^b^ Determined by STYH analysis sample distribution.

^c^ Joint *χ*^2^-test for the interaction term between food insecurity and food environment dimension.

STYH, Speak to Your Health.

## Discussion

Using self-report data from a 2015 cross-sectional cohort of adults in Flint, Michigan, individual-level food insecurity was associated with poor mental health, which is consistent with evidence from communities worldwide.^3^ In addition, mental health status tended to be better for elderly persons, married individuals, and those without any measured comorbidities. In addition, this study suggests that proximate access to vegetables and fruits may moderate the association between food insecurity and poor mental health. Furthermore, while other studies have used less precise metrics for representing the nutrition environment, this is the first to incorporate the GIS-based NEMS-S assessment as a combined community-consumer food environment variable in a study of mental health outcomes.

Psychosocial strain, malnutrition, and poor diet quality are underlying pathways by which food insecurity among adults may lead to poor mental health status through chronic systemic inflammation.^9,37^ The stress and emotional burden characteristic of food insecurity enhances the innate immune response and increases production of inflammatory cytokines,^38^ which are implicated in poor mental health outcomes such as depression.^39^

Interventions targeting malnutrition due to food insecurity may ameliorate mental health disparities. Nutrient-poor, calorically dense foods are often more affordable than healthier options, which results in poorer diet quality among those who are food insecure.^9^ Previous work demonstrates the influence of diet quality on mental health through inflammatory mechanisms.^10^ Evidence indicates that diet quality improves when a supermarket is introduced to an area that previously had fewer stores providing access to food resources.^40^

### Limitations

Despite the novel findings and innovative approach taken in this study, there were several limitations. First, the cross-sectional design of the STYH survey prevented us from assessing the temporal nature of observed relationships. This is important as the association between food insecurity and mental health is likely to some degree bidirectional. Depressive disorders and poor mental health are economically burdensome and may compete for the same resources used to maintain food security.^41^

A proportion of the study population was recruited through word of mouth, which limits the ability to extrapolate results to the general population. However, this snowballing approach is useful for recruiting difficult to reach participants, such as individuals who are of low income or are persons of color, and even probability sampling has inherent bias.^42^ Furthermore, the unique context of the Flint Water Crisis might limit generalizability of our findings, so future research should explore this relationship in other contexts. While the observed associations were adjusted for several demographic, socioeconomic, and health-related covariates, residual confounding by unobserved variables cannot be ruled out.

Measurement of food insecurity available in the STYH survey may also be problematic. To adequately measure the four dimensions necessary to identify individual food insecurity (i.e., quality, quantity, psychological acceptability, and social acceptability), the USDA 10–18 item household-level Food Security Survey Module (FSSM) is recommended.^6^ Evidence indicates, however, that even a two-item screener retains sufficient sensitivity, specificity, and validity.^43^ In addition, descriptive statistics within the study sample demonstrate bivariate associations of food insecurity—as assessed in this study—with demographic and socioeconomic factors associated with widely accepted measures of food insecurity.^4^ Food insecurity was more common among those who identified as a person of color, younger age groups, and those who were single. Food insecurity was also more common among those with low educational attainment and those who had extreme difficulty meeting household needs. These associations are consistent with evidence using the FSSM to identify food insecurity.^9^ Furthermore, the measure of food insecurity used in this analysis was not associated with the quality of local food environment—indicating that our measurement of individual food insecurity is assessing a construct distinct from objectively measured food access. The degree to which food insecurity, as measured by STYH, correlates with more comprehensive measures of food insecurity requires further study. Validation of study findings would also benefit from replication within data sources that use the FSSM to assess food insecurity.

Poor mental health differs from diagnosed psychopathology. Indeed, those with a diagnosed mental illness may report good mental health due to self-management and therapeutic treatment. Thus, additional research is needed to determine whether observed findings are similar when considering mental illnesses such as major depression.

Finally, use of the adapted NEMS-S survey and the spatial analysis technique employed to derive local food environment scores has limitations. While the NEMS-S has been validated in previous studies,^44^ data collection for NEMS-S was carried out in 2016, 1 year after 2015 STYH survey data were collected. Thus, it is possible that the food environment changed during this lag period. Second, although it is the recommended method for understanding exposure or access in geographic research,^45^ the use of kernel density remains underdeveloped, and does not perfectly represent an individual's food environment.

## Conclusion

The overall objective of this study was to examine associations of local food environment and food insecurity with mental health among adult residents of Flint, Michigan. Food insecurity was positively associated with poor mental health, and this relationship was moderated by one's local food environment. Among those with greater access to fruits and vegetables, the relationship of food insecurity and poor mental health was weaker. Findings suggest that living in an area with access to better food quality may blunt the negative impact of food insecurity on mental health. Longitudinal data are needed to determine whether increasing accessing to fruits and vegetables within communities at risk of food insecurity could improve mental health of residents.

## Abbreviations Used

CI: confidence interval
FSSM: Food Security Survey Module
NEMS-S: Nutrition Environment Measures Survey in Stores
OR: odds ratio
STYH: Speak to Your Health

## References

[1] Mathers CD, Loncar D Projections of global mortality and burden of disease from 2002 to 2030. PLoS Med. 2006;3:e442 17132052 10.1371/journal.pmed.0030442 PMC1664601

[2] Weaver LJ, Hadley C Moving beyond hunger and nutrition: a systematic review of the evidence linking food insecurity and mental health in developing countries. Ecol Food Nutr. 2009;48:263–284 21883069 10.1080/03670240903001167

[3] Jones AD Food insecurity and mental health status: a global analysis of 149 countries. Am J Prev Med. 2017;53:264–273 28457747 10.1016/j.amepre.2017.04.008

[4] Coleman-Jensen A, Rabbitt MP, Gregory CA, et al. Household Food Security in the United States in 2017. Alexandria, VA: U.S. Department of Agriculture, Economic Research Service, 2018

[5] Bickel G, Nord M, Price C, et al. *Guide to Measuring Household Food Security*. Alexandria, VA: U.S. Department of Agriculture, Food and Nutrition Service, 2000. Available at www.fns.usda.gov/sites/default/files/FSGuide_0.pdf Accessed 421, 2015

[6] Radimer KL, Kathy L Measurement of household food security in the USA and other industrialised countries. Public Health Nutr. 2002;5:859–864 12633509 10.1079/PHN2002385

[7] Ballard TJ, Kepple AW, Cafiero C The Food Insecurity Experience Scale. Development of a Global Standard for Monitoring Hunger Worldwide. Rome: Food and Agriculture Organization of the United Nations, 2013 Available at www.fao.org/3/a-as583e.pdf Accessed 925, 2018

[8] Hanson KL, Connor LM Food insecurity and dietary quality in US adults and children: a systematic review. Am J Clin Nutr. 2014;100:684–692 24944059 10.3945/ajcn.114.084525

[9] Bergmans RS, Palta M, Robert SA, et al. Associations between food security status and dietary inflammatory potential within lower-income adults from the United States National Health and Nutrition Examination Survey (NHANES), cycles 2007 to 2014. J Acad Nutr Diet 2018;118:994–1005 29452975 10.1016/j.jand.2017.12.003 PMC5971121

[10] Bergmans RS, Malecki KM The association of dietary inflammatory potential with depression and mental well-being among US adults. Prev Med. 2017;99:313–319 28342730 10.1016/j.ypmed.2017.03.016 PMC5484161

[11] Jung A, Spira D, Steinhagen-Thiessen E, et al. Zinc deficiency is associated with depressive symptoms—results from the Berlin Aging Study II. J Gerontol Ser A. 2017;72:1149–1154 10.1093/gerona/glw218 27789618

[12] Colón-Ramos U, Monge-Rojas R, Stevenson TR, et al. How do African-American caregivers navigate a food desert to feed their children? A photovoice narrative. J Acad Nutr Diet. 2018;118:2045–2056 29934282 10.1016/j.jand.2018.04.016

[13] Mayer VL, McDonough K, Seligman H, et al. Food insecurity, coping strategies and glucose control in low-income patients with diabetes. Public Health Nutr. 2016;19:1103–1111 26328922 10.1017/S1368980015002323 PMC10270824

[14] Lazarus RS, Folkman S Stress, Appraisal and Coping. New York: Springer, 1984

[15] Cohen BE Community Food Security Assessment Toolkit. Washington, DC: US Department of Agriculture, Economic Research Service, 2002

[16] Beaumont J, Lang T, Leather S, et al. Report from the Policy Sub-group to the Nutrition Task Force Low Income Project Team of the Department of Health. Radlett, Hertfordshire: Institute of Grocery Distribution, 1995

[17] Walker RE, Keane CR, Burke JG Disparities and access to healthy food in the United States: a review of food deserts literature. Health Place. 2010;16:876–884 20462784 10.1016/j.healthplace.2010.04.013

[18] Sadler RC, Lafreniere DJ Racist housing practices as a precursor to uneven neighborhood change in a post-industrial city. Hous Stud. 2017;32:186–208

[19] Shaver ER, Sadler RC, Hill AB, et al. The Flint Food Store Survey: combining spatial analysis with a modified Nutrition Environment Measures Survey in Stores (NEMS-S) to measure the community and consumer nutrition environments. Public Health Nutr. 2018;21:1474–1485 29361993 10.1017/S1368980017003950 PMC6941423

[20] Goodnough A, Atkinson S A Potent Side Effect to the Flint Water Crisis: Mental Health Problems. The New York Times. Published April 30, 2016. Available at https://www.nytimes.com/2016/05/01/us/flint-michigan-water-crisis-mental-health.html Accessed 312, 2018

[21] Pieper KJ, Tang M, Edwards MA Flint water crisis caused by interrupted corrosion control: investigating “ground zero” home. Environ Sci Technol. 2017;51:2007–2014 28145123 10.1021/acs.est.6b04034

[22] Shivappa N, Steck SE, Hurley TG, et al. Designing and developing a literature-derived, population-based dietary inflammatory index. Public Health Nutr. 2014;17:1689–1696 23941862 10.1017/S1368980013002115 PMC3925198

[23] Kruger DJ, Shirey L, Morrel-Samuels S, et al. Using a community-based health survey as a tool for informing local health policy. J Public Health Manag Pract. 2009;15:47–53 19077594 10.1097/PHH.0b013e3181903b9d

[24] U.S. Census Bureau QuickFacts: Flint city, Michigan. Available at https://www.census.gov/quickfacts/fact/table/flintcitymichigan/PST040217#viewtop Accessed 927, 2018

[25] Cook JT, Black M, Chilton M, et al. Are food insecurity's health impacts underestimated in the U.S. population? Marginal food security also predicts adverse health outcomes in young U.S. children and mothers. Adv Nutr Int Rev J. 2013;4:51–61 10.3945/an.112.003228 PMC3648739 23319123

[26] Ahmad F, Jhajj AK, Stewart DE, et al. Single item measures of self-rated mental health: a scoping review. BMC Health Serv Res. 2014;14:398 25231576 10.1186/1472-6963-14-398 PMC4177165

[27] Levinson D, Kaplan G What does self rated mental health represent. J Public Health Res. 2014;3: 287 25553310 10.4081/jphr.2014.287 PMC4274494

[28] Mawani FN, Gilmour H Validation of self-rated mental health. Health Rep. 2010;21:61–75 20973435

[29] McKinnon RA, Reedy J, Handy SL, et al. Measuring the food and physical activity environments: shaping the research agenda. Am J Prev Med. 2009;36(4 Suppl):S81–S85 19285213 10.1016/j.amepre.2009.01.003

[30] Terrell GR, Scott DW Variable kernel density estimation. Ann Stat. 1992;20:1236–1265

[31] Mau MK, Wong KN, Efird J, et al. Environmental factors of obesity in communities with native Hawaiians. Hawaii Med J. 2008;67:233 18853897 PMC2586007

[32] Jiao J, Moudon AV, Ulmer J, et al. How to identify food deserts: measuring physical and economic access to supermarkets in King County, Washington. Am J Public Health. 2012;102:e32–e39 22897554 10.2105/AJPH.2012.300675 PMC3490650

[33] Rivera M, Rovira P, Gutierrez B, et al. Comorbid medical conditions in individuals with major psychiatric disorders. Eur Neuropsychopharmacol. 2017;27:S396–S397

[34] Tarasuk V Health implications of food insecurity. In: Social Determinants of Health: Canadian Perspectives. Edited by Raphael D, 321st-342nd ed. Toronto, Canada: Canadian Scholars' Press, Inc, 2004, pp. 187–200

[35] SAS Institute, Inc., SAS Software Verrsion 9.4. Cary, NC: SAS Institute Inc., 2013

[36] SAS Institute. Support/Samples & SAS Notes (Usage Note 24455): Estimating an odds ratio for a variable involved in an interaction. Published 2013. Available at http://support.sas.com/kb/24/455.html Accessed 312, 2018

[37] Seligman HK, Schillinger D Hunger and socioeconomic disparities in chronic disease. N Engl J Med. 2010;363:6–9 20592297 10.1056/NEJMp1000072

[38] Maes M, Song C, Lin A, et al. The effects of psychological stress on humans: increased production of pro-inflammatory cytokines and Th1-like response in stress-induced anxiety. Cytokine. 1998;10:313–318 9617578 10.1006/cyto.1997.0290

[39] Miller AH, Raison CL The role of inflammation in depression: from evolutionary imperative to modern treatment target. Nat Rev Immunol. 2016;16:22–34 26711676 10.1038/nri.2015.5 PMC5542678

[40] Dubowitz T, Ghosh-Dastidar M, Cohen DA, et al. Diet and perceptions change with supermarket introduction in a food desert, but not because of supermarket use. Health Aff (Millwood). 2015;34:1858–1868 26526243 10.1377/hlthaff.2015.0667 PMC4977027

[41] Lépine J-P, Briley M The increasing burden of depression. Neuropsychiatr Dis Treat. 2011;7(Suppl 1):3–7 21750622 10.2147/NDT.S19617 PMC3131101

[42] Sadler GR, Lee H-C, Seung-Hwan Lim R, et al. Recruiting hard-to-reach United States population sub-groups via adaptations of snowball sampling strategy. Nurs Health Sci. 2010;12:369–374 20727089 10.1111/j.1442-2018.2010.00541.x PMC3222300

[43] Hager ER, Quigg AM, Black MM, et al. Development and validity of a 2-item screen to identify families at risk for food insecurity. Pediatrics. 2010;126:e26–e32 20595453 10.1542/peds.2009-3146

[44] Glanz K, Sallis JF, Saelens BE, et al. Nutrition Environment Measures Survey in stores (NEMS-S): development and evaluation. Am J Prev Med. 2007;32:282–289 17383559 10.1016/j.amepre.2006.12.019

[45] Kestens Y, Lebel A, Chaix B, et al. Association between activity space exposure to food establishments and individual risk of overweight. PLoS One. 2012;7:e41418 22936974 10.1371/journal.pone.0041418 PMC3425582

